# Editorial: Human Microbiome: Symbiosis to Pathogenesis

**DOI:** 10.3389/fmicb.2021.605783

**Published:** 2021-02-17

**Authors:** Learn-Han Lee, Sunny Hei Wong, Siok-Fong Chin, Vishal Singh, Nurul-Syakima Ab Mutalib

**Affiliations:** ^1^Novel Bacteria and Drug Discovery (NBDD) Research Group, Microbiome and Bioresource Research Strength (MBRS), Jeffrey Cheah School of Medicine and Health Sciences, Monash University Malaysia, Subang Jaya, Malaysia; ^2^Li Ka Shing Institute of Health Sciences, Department of Medicine and Therapeutics, The Chinese University of Hong Kong, Shatin, Hong Kong; ^3^UKM Medical Molecular Biology Institute (UMBI), Universiti Kebangsaan Malaysia, Kuala Lumpur, Malaysia; ^4^Department of Nutritional Sciences, The Pennsylvania State University, State College, PA, United States

**Keywords:** human microbiome, symbiosis, pathogenesis, fecal microbiome transplant, gut microbiome

## Introduction

Researchers have come a long way in microorganism research since the first discovery of microbes as seen through an ingenious hand-held microscope over 300 years ago. In late 1600, the father of microbiology, Antonie van Leeuwenhoek, came up with his microscope design and observed tiny moving objects. He named the tiny objects animalcules (Leeuwenhoek, 1963; Smit and Heniger, 1975; Gest, 2004). Since then, the number of studies on microbes began to increase, opening up for a new horizon for research—the roles of microbes living not just in the environment but also throughout the human body.

The human microbiome consists of microbial communities, such as bacteria, virus, and fungi, which resides in and on our bodies. As the gut microbiome shifts toward a mature state after 2 years of human growth, numerous factors, such as drug use (e.g., antibiotics), dietary intake, and nutritional supplements, can lead to bacterial composition differences (Cho and Blaser, 2012; Voreades et al., 2014).

In this Research Topic, the Human Microbiome: Symbiosis to Pathogenesis, a total of 27 articles have been published, covering several exciting aspects that highlight the role of microbiota in humans, how microbes interact with the host, and how they subsequently contribute to the pathogenesis of chronic inflammatory diseases as well as metabolic disorders. Based on the gathered information, some research groups have also presented the potential of exploiting these data to design effective preventive and therapeutic options targeting specific bacterial communities.

## Revolutionary Tools of Scientific Research: More Sensitive, Advanced, and Accurate Methods for Microbiome Research

On top of its current affordable pricing, next-generation sequencing speeds up the bacterial identification processes (by bypassing the need for culturing process) while providing enormous valuable information when combined with big data from other analytical techniques such as flow cytometry and gas or liquid chromatographic methods (Lagier et al., 2012; Escobar-Zepeda et al., 2015; Fouhy et al., 2015; Wang et al., 2015; Jovel et al., 2016; Lin et al., 2019; Osman et al.). A recent study has proposed a new, revised estimated ratio of 1:1 bacterial to human cells after studying the human microbiome thoroughly a few years ago (Sender et al., 2016). Specifically, the colon contains ~10 trillion cells, or 33% of the total bacterial cells in the human body, making it a site with the most microbes compared to other gastrointestinal system sites. Interestingly, Dong et al. shared their findings in this Research Topic, where they observed specific site-preferable bacterial signatures in the oral cavity and other parts of the gastrointestinal tract. These findings are significant in serving as a form of “baseline” to improving the understanding of the microbiome in human health.

## How Does the Microbiome Reflect One's State of Health?

Till now, the relationship between humans and microbes appears to be rather complicated; one may even refer to it as a dramatic love-hate relationship as some can cause deadly infections while a portion of them improves the health of the host (Rosenstiel, 2013; Tomkovich and Jobin, 2016; Postler and Ghosh, 2017). As hosts, humans provide a habitat for microbes. In exchange, most microbes return the “favor” in a different form(s), including providing nutrients for the host by breaking down specific substrates (i.e., supporting the host's metabolism) and defending the host against harmful, opportunistic pathogens via habitat colonization and immunomodulatory responses (Iacob et al., Laville et al., Rodionov et al., Sharma et al., Shin et al.). Iacob et al. discussed the importance of the intestinal microbiome in maintaining barrier integrity while promoting and preserving immune homeostasis during intestinal infections. Whether or not the infant's gut is sterile or without microorganisms at birth remains a hot debate among the scientific community. The infant's gut microbiota begins to flourish within hours after birth and starts to stabilize upon reaching 2 years of age (Backhed et al., 2015; Bokulich et al., 2016; Yang et al., 2016; Chong et al., 2018; Mohammadkhah et al., 2018). Several factors lead to the microbiome's differences, including delivery mode, breastfeeding history, and drug use (e.g., direct antibiotic use, or mother antenatally). For example, infants delivered via cesarean delivery who lack exposure to the vaginal microbiome have a higher risk of developing asthma, autoimmune diseases, and metabolic diseases. Thus, it is now widely recognized that exposure to the vaginal microbiome of the mother or “vaginal seeding” assist in the formation of a healthy gut microbiome and “priming” the immune system of the infant (Dominguez-Bello et al., 2010; Ferretti et al., 2018; Stinson et al., 2018; Zhu et al.). Zhu et al. challenged the idea of a “sterile” healthy uterine cavity for pregnancy and discussed that bacterial colonization does occur during pregnancy. Even though a metagenomic study based on the 16S rRNA gene revealed certain bacterial groups, these microbes were either non-viable or rarely culturable. On the contrary, the team managed to recover, culture, and identify bacteria from the placenta, which led to the speculation that the bacteria recovered from amniotic fluid might spread through other locations (e.g., blood or placenta). Be that as it may, findings from Liu et al. further supported the notion that the delivery mode affects more than just the infant's microbiome. The team observed a rather peculiar change in the maternal urinary microbiome after cesarean delivery: many beneficial bacteria (including those affiliated with the phylum *Firmicutes*) dramatically reduced with an increment of potentially pathogenic bacterial populations such as *Pseudomonas* spp. (under the phylum *Proteobacteria* and family *Pseudomonadaceae*). These results suggest these microbial populations' potential, which may serve as risk markers for clinicians to consider protecting these mothers from infectious diseases.

Gut dysbiosis can directly affect gastrointestinal health (Zuo and Ng, Wang et al., Li et al., Guo et al., Bodkhe et al., Lu et al., Dinakaran et al., Chong et al., Jacouton et al., Deng et al.). The dysbiosis can occur due to overgrowth or invasion of pathogens in the gut, compromising the gut epithelial barrier integrity and exacerbating inflammation (Natividad and Verdu, 2013; Zhang et al., 2015; Wong et al., 2017). As chronic inflammation in the colon can lead to colon cancer development, Osman et al. reviewed the methods and databases available to help determine dysbiosis and summarized a list of bacteria associated with colorectal cancer based on previous literature. On the other hand, Wang et al. presented a piece of evidence suggesting the gut microbiome's causative role in non-alcoholic fatty liver disease. Gnotobiotic mice that received gut microbiota from genetically obese human donors developed liver macrovesicular steatosis with a higher concentration of hepatic triglyceride and cholesterol. These symptoms were not observed in those receiving gut microbiota from a genetically obese human donor after a dietary weight loss program. Similarly, Deng et al. also used gnotobiotic mice in their study, which showed that two groups of mice receiving gut microbiota from a child before and after dietary intervention (i.e., the WTP diet, composed of whole grains, traditional Chinese medicine food, and prebiotics) resulted in very different physiological phenotypes, each of which was similar to the donor's phenotype. The group analyzed differentially expressed (DE) miRNAs and genes in the colon and liver, which subsequently suggested the causative role of gut dysbiosis in obesity. Dinakaran et al. studied an array of full-thickness colon specimens of inflammatory bowel disease (IBD) patients. They found a significant difference in dysbiosis between African Americans compared to Caucasian patients along with a higher abundance of pathogenic bacteria at the diseased site than adjacent healthy colon specimens.

Apart from colon cancer and liver diseases, Chong et al. performed a thorough literature search and gathered necessary information highlighting the association between gut dysbiosis and irritable bowel syndrome (IBS). As a multifactorial disease, IBS is a chronic, functional bowel disorder that requires long-term management, including dietary and lifestyle changes (Selvaraj et al., 2020). The most striking part was the presence of co-morbid disorders among these patients, particularly anxiety and depression. There is still an apparent lack of explanations as to how microbes in the gut communicate with neurons in the brain that drives behavioral or emotional changes, but more clues are emphasizing the importance of the gut–brain axis (Foster and Neufeld, 2013; Kennedy et al., 2014; Lee et al., 2018, 2019; Quigley, 2018; Johnson et al., 2020). Coretti et al. observed a substantial increase of *Bacteroidetes* and *Proteobacteria*, with a decrease of *Actinobacteria* in young children (aged 2–4 years of age) with autism spectrum disorder (ASD). The same team examined fecal short-chain fatty acid (SCFA) levels and discovered a significant increase of butyrate in ASD patients compared to healthy controls. Even though their levels were still within the normal range, the study pointed out that the over-representation of butyrate-producing bacteria can potentially result in neurologic and neuropsychiatric disorders.

Besides supporting the host's metabolism, microbes produce a wide array of beneficial compounds, including antibiotics and certain SCFAs that can function as “signaling molecules” and modulate the host's immune response (Ganapathy et al., 2013). Zuo and Ng published a review under this Research Topic, emphasizing the role of the gut microbiome in IBD. They indicated that a low intake of dietary fiber and low levels of SCFAs were associated with the development of IBD. Aside from that, the gut microbiome's stability and composition can be influenced by the host's dietary intake and dietary supplements like probiotics and prebiotics, emphasizing the importance of changing the host's eating behavior to restore health by boosting the balance in microbial composition.

## Microbiome Changes as Biomarkers and Potential Treatment Target

Microbes within us could be the answer to identifying and resolving a plethora of health conditions. Instead of using invasive methods such as blood drawing or endoscopy examination, some teams have advocated the idea of screening fecal microbiome to identify specific bacterial signatures that may be used as a biomarker or even prognostic marker. Lu et al. discussed the unique gut microbial characteristics of liver recipients with abnormal and normal liver functions. The study described the fecal microbiome index measuring specific alterations of *Staphylococcus* and *Prevotella* spp. that could be used to distinguish liver recipients with abnormal and normal liver function. These findings could then help clinicians make subsequent decisions to ensure the swift recovery of liver function and restore the intestinal microbiota in patients after transplantation.

One of the easiest ways to manipulate the gut microbiome is via alterations in dietary intake. The concept of pre-and probiotics has always been under the limelight in the food industry. Probiotics are microbes that promote health effects in the host (when administered in adequate amounts), while prebiotics consists of non-digestible food or compounds that can be metabolized by microbes and grant beneficial physiological effects to the host via modulation of microbial composition or activity (Ziemer and Gibson, 1998; Martín and Langella, 2019). For instance, *Lactococcus* spp. is a frequently used probiotic in dairy products, and they can regulate the immune response via direct production of cytokines or induction of cytokines in immune cells (Del Carmen et al., 2011; Xu and Kong, 2013; Yin et al., 2014). Jacouton et al. presented that *Lactobacillus casei* BL23 confers antitumor activity against colorectal cancer via the regulation of natural killer cell activity and stimulation of IL-2 production. Undeniably, further in-depth investigations into the stability and persistence of beneficial microbes via supplementation and prebiotics can improve host-microbe relationships. Researchers can apply this knowledge to therapeutic and preventive measures strategy against diseases by reversing dysbiosis in the microbiome and improving humans' overall health (Ma et al., 2020; Yue et al., 2020). Rodionov et al. and Sharma et al. emphasized the idea of the dietary response of gut microbial communities when it came to designing treatment for dysbiosis-related syndromes and diseases via rational nutritional supplementation. Rodionov et al. utilized genomics and a predictive modeling approach to study eight B vitamins, queuosine utilization, and sharing capabilities in the gut microbiome. Likewise, experimental studies by Sharma et al. supported the hypothesis of B vitamin sharing among gut microbes as the relative abundance of auxotrophic species remained unchanged in the absence of B-vitamins or significant excess. In summary, understanding the interactions between microbe–microbe interactions is just as vital as host–microbe interactions when it comes to formulating preventive and therapeutic strategies targeting microbiome restoration.

Besides that, FMT is a procedure that delivers specially packed stool material from healthy donors to the recipient to improve health conditions by restoring the balance in the gut microbial community (Aroniadis and Brandt, 2013). FMT utilization began during the Chinese civilization: a traditional Chinese medicine doctor cured patients who had food poisoning and severe diarrhea by consuming “yellow soup” containing human fecal suspension (Zhang et al., 2012; Shi and Yang, 2018). In modern days, FMT is used to treat patients with multiple recurrent *Clostridium difficile* infections and who failed to respond to conventional antibiotics treatment (Liubakka and Vaughn, 2016; Nagy, 2018; Nicholson et al., 2020; Perler et al., 2020; Li et al.). Li et al. examined fecal samples from children with recurrent *C. difficile* infection (RCDI) after undergoing FMT. They noticed a shift in microbiome composition with similarity leaning toward the donor and healthy control group, possibly with a better gut health state. Even though four of them received more than a single FMT session as they did not exhibit clinical improvement after the first FMT, they still achieved remission after multiple FMT.

Other than the gut microbiomes, there is potentially increasing attention placed on studying the microbiomes of organs other than the gastrointestinal system for humanity's benefits, especially for the development of biomarkers. Under this Research Topic, there are a total of six articles exploring the microbiome of other organs such as the nasal, vaginal, urinary, and prostate microbiomes (Balan et al., Zhu et al., Liu et al., Gliniewicz et al., Ma et al., Camelo-Castillo et al.). Ma et al. compared the microbiome profiles of prostatic fluid samples from prostate cancer patients with healthy controls. Typically, detection of prostate cancer is performed using invasive procedures like biopsies and the measurement of prostate-specific antigen, which frequently leads to misdiagnosis due to its high sensitivity and low specificity (Etzioni et al., 2002; Welch and Albertsen, 2009; Kasivisvanathan et al., 2018). This pilot study presented by Ma et al. demonstrated a lower microbial diversity in the prostate fluid of prostate cancer patients compared to healthy controls. Camelo-Castillo et al. compared the nasopharyngeal microbiota of children with invasive pneumococcal disease (IPD) to matched controls by performing a principal coordinate analysis. While pointing out that specific microbiota profiles should be studied thoroughly as potential biomarkers for IPD or asymptomatic colonization, the study emphasized that identifying beneficial bacteria through this type of study could help fight against pneumococcal infections, possibly by integrating these microorganisms in a probiotic formula. Having that said, these studies serve as an essential steppingstone in identifying specific bacterial species as novel diagnostic biomarkers. They offer an exciting perspective to consider capitalizing on their roles in different diseases to formulate better therapeutic and prevention strategies in the nearest future.

## Conclusion

With the increasing ability to understand microbial metabolic and functional capabilities, the microbiome's contribution to human health and various diseases is becoming more evident. Researchers are attempting to untangle the complicated relationships between microbes and humans as the host. More work is needed to move toward personalized medicine by restoring balance in the gut microbiome, regardless of population replacement via FMT or introduction of prebiotics or probiotics. Just as insinuated by Bodkhe et al., it might be worthwhile to investigate the microbiome of close relatives of patients suffering heritable diseases such as celiac disease. The investigation may give a better overview of alterations in the microbiome during disease progression or transition. Given the complexity of interactions between host-microbe and microbe-microbe, defining the “baseline” for a healthy gut microbiome seems like the next hurdle for everyone in microbiome research. Nonetheless, the Research Topic's findings strengthen the current opinion on dysbiosis and human health and suggest the importance of seeking an association between different microbiomes in the human body. Understanding the symbiotic relationships between the human host and microbiome will guarantee success in the restoration of the microbiome for disease prevention, management, and treatment.

## Author Contributions

L-HL, SHW, and N-SAM wrote the editorial. L-HL, SHW, S-FC, VS, and N-SAM participated in reviewing, editing, and proofreading of the editorial. L-HL, SHW, and N-SAM conceptualized the project. All authors read and approved the final editorial.

## Conflict of Interest

The authors declare that the research was conducted in the absence of any commercial or financial relationships that could be construed as a potential conflict of interest.
